# Pneumococcal pneumonia prevalence among adults with severe acute respiratory illness in Thailand - comparison of Bayesian latent class modeling and conventional analysis

**DOI:** 10.1186/s12879-019-4067-3

**Published:** 2019-05-15

**Authors:** Ying Lu, Lawrence Joseph, Patrick Bélisle, Pongpun Sawatwong, Anchalee Jatapai, Toni Whistler, Somsak Thamthitiwat, Wantana Paveenkittiporn, Supphacoke Khemla, Chris A. Van Beneden, Henry C. Baggett, Christopher J. Gregory

**Affiliations:** 1 Division of Global Health Protection, Thailand Ministry of Public Health–US Centers for Disease Control and Prevention Collaboration, Nonthaburi, Thailand; 2 Department of Epidemiology and Biostatistics, McGill University, Montreal, Canada; 3 Centre Hospitalier de l’Universite de Montreal,Montreal, Montreal, Canada; 4 Office of Public Health, Regional Development Mission for Asia, US Agency for International Development, Bangkok, Thailand; 5 Department of Medical Sciences, Ministry of Public Health, National Institute of Health, Nonthaburi, Thailand; 6 Nakhon Phanom Provincial Hospital, Ministry of Public Health, Nakhon Phanom, Thailand; 7 Division of Bacterial Diseases, National Center for Immunization and Respiratory Disease, US Centers for Disease Control and Prevention, Atlanta, GA USA; 8 Division of Global Health Protection, US Centers for Disease Control and Prevention, Atlanta, GA USA; 9 Present affiliation: Division of Vector—Borne Diseases, US Centers for Disease Control and Prevention, Fort Collins, CO USA

**Keywords:** Pneumonia etiology, Pneumococcal pneumonia, Real-time polymerase chain reaction test, Urine antigen test, Pneumococcal density, Cycle threshold, Bayesian latent class

## Abstract

**Background:**

Determining the etiology of pneumonia is essential to guide public health interventions. Diagnostic test results, including from polymerase chain reaction (PCR) assays of upper respiratory tract specimens, have been used to estimate prevalence of pneumococcal pneumonia. However limitations in test sensitivity and specificity and the specimen types available make establishing a definitive diagnosis challenging. Prevalence estimates for pneumococcal pneumonia could be biased in the absence of a true gold standard reference test for detecting *Streptococcus pneumoniae*.

**Methods:**

We conducted a case control study to identify etiologies of community acquired pneumonia (CAP) from April 2014 through August 2015 in Thailand. We estimated the prevalence of pneumococcal pneumonia among adults hospitalized for CAP using Bayesian latent class models (BLCMs) incorporating results of real-time polymerase chain reaction (qPCR) testing of upper respiratory tract specimens and a urine antigen test (UAT) from cases and controls. We compared the prevalence estimate to conventional analyses using only UAT as a reference test.

**Results:**

The estimated prevalence of pneumococcal pneumonia was 8% (95% CI: 5–11%) by conventional analyses. By BLCM, we estimated the prevalence to be 10% (95% CrI: 7–16%) using binary qPCR and UAT results, and 11% (95% CrI: 7–17%) using binary UAT results and qPCR cycle threshold (Ct) values.

**Conclusions:**

BLCM suggests a > 25% higher prevalence of pneumococcal pneumonia than estimated by a conventional approach assuming UAT as a gold standard reference test. Higher quantities of pneumococcal DNA in the upper respiratory tract were associated with pneumococcal pneumonia in adults but the addition of a second specific pneumococcal test was required to accurately estimate disease status and prevalence. By incorporating the inherent uncertainty of diagnostic tests, BLCM can obtain more reliable estimates of disease status and improve understanding of underlying etiology.

**Electronic supplementary material:**

The online version of this article (10.1186/s12879-019-4067-3) contains supplementary material, which is available to authorized users.

## Background

An estimated 2.5 to 3 million pneumonia deaths occurred worldwide in 2010 [1]. Understanding the etiology of pneumonia is essential to guiding prevention strategies, improving clinical management, and minimizing the development of drug-resistance [2]. Pneumococcus is among the most important etiologies of hospitalized community-acquired pneumonia (CAP) among adults [3–6], but demonstrating *Streptococcus pneumoniae* as the etiology of pneumonia is challenging. In part this is because specimens from the site of infection (such as lung aspirates) are rarely collected nowadays for either clinical or research purposes. Pneumonia diagnosis currently tests largely on specimens not obtained directly from the lung (for example, blood, nasopharyngeal or oropharyngeal samples, urine, or induced sputum) which have imperfect sensitivity and specificity due to both the manner of collection and inherent to existing diagnostic tests such as culture or polymerase chain reaction (PCR) testing, despite advances in laboratory technology [6, 7].

Because it is rarely possible to definitively confirm pneumococcal pneumonia using existing diagnostic methods, this status is considered a “latent” variable. Latent class models (LCM) can link the latent variable with diagnostic tests results. This method has been used to estimate prevalence of pneumococcal pneumonia among adults with CAP in the United States (U.S.) and Kenya, incorporating results from multiple tests including PCR assays conducted on specimens from the upper respiratory tract or lung aspirate [2, 8]. Cycle threshold (Ct) values obtained from PCR are typically converted to a binary (positive or negative) result before further statistical analysis. However, when continuous or semi-quantitative test results such as Ct values are converted to a binary result, any potential association with the density of a detected pathogen is lost. The colonization density of *S. pneumoniae* has been shown in some but not all previous studies [9–13] to be associated with pneumococcal pneumonia.

During 2014 and 2015, the Thailand Ministry of Public Health (MOPH) and the U.S. Centers for Disease Control and Prevention (CDC) participated in a multicenter case-control study of the potential etiology of CAP among adults. In this study, real-time polymerase chain reaction (qPCR) testing for 16 viral and bacterial pathogens using the TaqMan array card (TAC) was performed on specimens collected from the upper respiratory tract. Similar to previous studies, *S. pneumoniae* was commonly detected but detection of *S. pneumoniae* from the upper respiratory tract can represent colonization in the absence of pneumococcal pneumonia, particularly among children [14–17].

We estimated the prevalence of pneumococcal pneumonia among adults aged 18 years and older who were hospitalized for CAP in Thailand during 2014 and 2015 using a Bayesian latent class model (BLCM) to incorporate Ct results of semi-quantitative pneumococcal qPCR testing of upper respiratory specimens and a second, more specific assay for *S. pneumoniae*, the urine antigen test (UAT) [18]. In addition, we compared the estimates from BLCM to those obtained by using qPCR results as a qualitative variable and to conventional analyses that considered UAT as a reference test for pneumococcal pneumonia. Secondary objectives included estimation of individual-level probability of pneumococcal pneumonia and evaluation of the test performance of UAT and qPCR of upper respiratory specimens to detect pneumococcal pneumonia.

## Methods

### Study population and setting

We identified eligible case-patients among persons ≥18 years old hospitalized from April 2014 through August 2015 in one of four hospitals in Nakhon Phanom province in Northeast Thailand: Nakhon Phanom, Nakae, Srisongkharm, and Tautane. We defined CAP as illness meeting WHO’s global influenza surveillance definition for severe acute respiratory illness (SARI): an acute respiratory infection and history of fever or measured fever of ≥38 C° and cough with onset within the last 7 days requiring hospitalization [19]. A potential case was excluded if he/she had been hospitalized for any cause in the previous 14 days or had an episode of pneumonia or been previously enrolled in the study within the past 30 days. We randomly selected controls from dental clinics in these hospitals to represent the population in the same residential areas as case-patients. Controls were frequency matched by age group (18–49, 50–64 and ≥ 65 years old) and enrolled within 2 weeks of case enrollment in order to control for pathogen seasonality. A control was excluded if he/she had been hospitalized for any cause in the previous 14 days, had an episode of pneumonia or were previously enrolled in the study within the past 30 days, or had any current symptom of acute respiratory infection. To maximize case enrollment while maintaining the ability to detect a statistical difference among cases and controls for viral and bacterial etiologies given the limited number of TAC cards available, we selected a 5:2 case to control target ratio.

### Specimen and data collection

Nasopharyngeal (NP) and oropharyngeal (OP) swabs and a urine specimen were collected from all enrolled cases and controls. A standard case report form was completed to capture demographic characteristics, medical history, clinical signs and symptoms, date of illness onset, hospital course and outcome including receipt of antibiotics prior to specimen collection. NP/OP swabs were collected into 3 ml of universal transport medium (UTM) at the study sites, aliquoted, frozen and transported to the central laboratory where they were stored at − 70 °C. Total nucleic acid (TNA) extraction from 200 ul of NP/OP swabs UTM was performed using the NucliSens easyMAG system (BioMerieux, Marcy l’Etoile, France), with a final elution volume of 100 ul. The *S. pneumoniae* primers and probes on the TAC were against the autolysin (*LytA*) gene from previously published assays [20]. Briefly, 46 μl specimen TNA was used in the AgPath-ID One-Step RT-PCR kit, (Applied Biosystems, CA, USA). A no-template control and a positive control consisting of combined RNA transcripts, generated as previously described [21], were included on each TAC along with up to six patient specimens. Reaction mixtures were loaded into the individual portals, and the card was centrifuged twice at 335×g for 1 min and sealed to close the reaction wells. All TACs were run on an Applied Biosystems ViiA7 real-time PCR instrument (Life Technologies) using the following cycling conditions: 45 °C for 10 min, 94 °C for 10 min, 45 cycles of 94 °C for 30 s, and 60 °C for 60 s. The Ct value represents the number of amplification cycles needed before the pathogen-specific nucleic acid was detected. Therefore, higher Ct values indicate lower amounts of the target organism DNA in the extracted sample.

Urine was tested in the hospital laboratory by the Binax NOW® *S. pneumoniae* Antigen Card immunochromatographic test. Positive and negative controls provided by the manufacturer were run daily. Published estimates of the sensitivity and specificity of this assay from a meta-analysis on UAT performance are 0.75 (95% CI: 0.71–0.79) and 0.95 (95% CI: 0.92–0.98) [22].

### Statistical analysis

To estimate the prevalence of pneumococcal pneumonia in cases, both conventional analyses with UAT as the reference test and BLCMs without referencing a gold standard test were conducted.

#### Conventional analyses

For the primary conventional analysis, UAT was assumed to have 100% sensitivity and specificity; patients with positive UAT results were considered true pneumococcal pneumonia cases and those with negative UAT results were considered non-pneumococcal pneumonia cases. Prevalence was estimated using the proportion of UAT positive (a binomial proportion), and 95% confidence interval (CI) was calculated based on standard error of the binomial proportion. The mean Ct value among pneumococcal pneumonia cases was calculated as the mean of Ct values among UAT-positive patients with a positive qPCR result. The mean Ct value in non-pneumococcal pneumonia was calculated similarly among UAT-negative participants. We also conducted an adjusted conventional analysis to obtain a prevalence estimate adjusted by the reported sensitivity of UAT from previous studies [22]. The prevalence estimate from the primary conventional analysis model was divided by 75%. These analyses were done in RStudio (Version 1.0.136 –© 2009–2016 RStudio, Inc.)

#### Bayesian latent class modelling (BLCM)

BLCM models were run using either binary or continuous qPCR results; both models include UAT results. We used the model by Joseph et al. [23] to analyze qPCR results as a binary variable using the Bayes Diagnostic Tests program available from http://www.medicine.mcgill.ca/epidemiology/Joseph/software/Diagnostic-Testing.html. A model based on Weichenthal et al. [24] was created for utilizing qPCR Ct values. Parameters included in the models and methods for the selection of prior distributions of those parameters and sensitivity analyses are described in the Additional file 1: Appendix. We ran 20,000 iterations of the Gibbs sampler algorithm after burn-in of 5000 iterations. All model runs were checked for convergence using the convergence diagnostics provided by the WinBUGS v. 1.4.3 (Imperial College and MRC, UK, see the WinBUGS code we used, provided in the Appendix), via examination of the history plots of all parameters.

## Results

From April 2014 to August 2015, 5171 hospitalized patients were screened for CAP; among 462 eligible patients, 357 (77.3%) were enrolled. During the same period, 255 healthy individuals were screened as potential controls; 238 were eligible and 217 (91.2%) enrolled. Among case-patients, 45% (161/357) were male with a mean age of 62 years (standard deviation SD: 18 years). Controls were 37% (80/217) male with a mean age of 58 years (SD: 16 years).

**Fig. 1:**
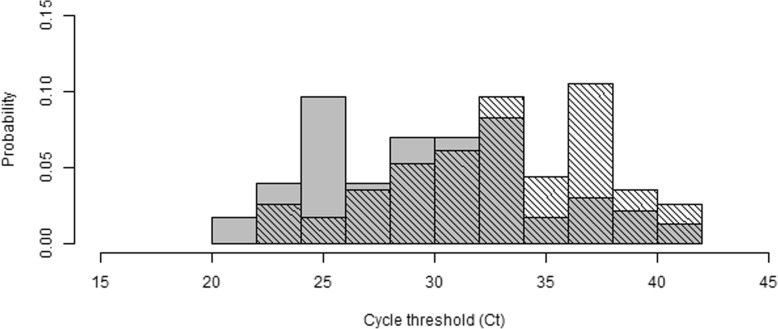
Distribution of pneumococcal PCR Ct values among adults hospitalized with community acquired pneumonia (*n* = 114) and qPCR positive controls (*n* = 57)*

Among 357 case-patients, 32% (114/357) had a NP/OP specimen positive for *S. pneumoniae* by qPCR compared to 26% (57/217) of controls. Of 353 case-patients also tested by UAT, 8% (27/353) were positive compared to 0.5% (1/217) of controls. While no controls were positive by both assays, 7.4% (26/353) of case-patients were UAT and qPCR positive (Table 1). In qPCR-positive samples, the mean Ct value among case-patients was lower (29.8 [SD: 4.9]) than that among controls (33.0 [SD: 4.8]); however, the distribution of case and control Ct values overlapped (Fig. 1). UAT-positive case-patients had a mean Ct value of 25.6 (SD: 3.3) compared to 31 (SD: 4.7) in UAT negative case-patients. Seventy-five percent (266/357) of case-patients received antibiotics prior to NP/OP swab; 35% (93/266) of these case-patients were qPCR positive, with a mean Ct value of 29.9 (SD: 4.7). Among 91 case-patients who did not receive antibiotics, 23% (21/91) were qPCR positive with a mean Ct value of 28.9 (SD: 5.7).

**Table 1:** *S. pneumoniae* detection by real-time polymerase chain reaction (qPCR) on nasopharyngeal and oropharyngeal specimens and urine antigen test (UAT) among case-patients with community-acquired pneumonia in Nakhon Phanom, Thailand, April 2014 – August 2015

		UAT		Total
		Positive	Negative	
qPCR	Positive	26	85	111 (31%)
	Negative	1	241	242
Total		27 (8%)	326	353*

*Among 357 cases, four cases did not have UAT test done

### Conventional analysis

In conventional analysis, prevalence of pneumococcal pneumonia among cases was 8% (95% CI: 5–11%). The positive predictive value (PPV) of UAT was 100%. The prevalence was 10% (95% CI: 7–15%) with the adjusted analysis, which considered UAT to be 75% sensitive (Table 2).

**Table 2:** Prevalence of pneumococcal pneumonia (PPN) in case-patients and performance of real-time polymerase chain reaction (qPCR) and urine antigen test (UAT): conventional analysis with UAT as a gold standard vs. Bayesian latent class models (BLCM) (*n* = 353)

	Conventional analyses Mean (95% CI)		BLCM Median (95% CrI)	
	Primary	Adjusted	Binary qPCR model	Continuous qPCR model
	27 UAT positives considered true positives	UAT sensitivity is 75% and specificity is 100%	qPCR and UAT	qPCR and UAT
Prevalence	8% (5–11%)	10% (7–15%)	10% (7–16%)	11% (7–17%)
Sensitivity of qPCR	96% (81–100%)		96% (83–100%)	
Specificity of qPCR	74% (69–79%)		75% (71–79%)	
PPV of qPCR	23% (16–32%)		31% (20–44%)	
Mean qPCR Ct among PPN	25.6 (24.2–26.9)			25.7 (24.6–27)
Mean qPCR among Non-PPN	31 (30–32)			32.4 (31.4–33.4)
Sensitivity of UAT	100% (assumed)	75% (published)	72% (53–87%)	67% (51–83%)
Specificity of UAT	100% (assumed)	100% (assumed)	99.5% (98.5–99.9%)	99.5% (98.6–99.9%)
PPV of UAT	100%	100%	94% (83–99%)	95% (83–99%)
Probability of PPV of UAT < 90%	0	0	18%	17%

### BLCM with qualitative qPCR results

The estimated pneumococcal pneumonia prevalence among case-patients was 10% (95% CrI: 7–16%) with a PPV for pneumococcal pneumonia given a positive qPCR of 31% (95% CrI: 20–44%). (Table 2). The specificity of UAT was estimated as 99.5% (95% CrI: 98.5–99.9%) and the sensitivity as 72% (95% CrI: 53–87%). The specificity of qPCR was estimated as 75% (71–79%) and the sensitivity as 96% (95% CrI: 83–100%). The PPV for pneumococcal pneumonia given a positive UAT was 94% (95% CrI: 83–99%).

### BLCM with continuous qPCR results

The prevalence of pneumococcal pneumonia in case-patients was estimated at 11% (95% CrI: 7–17%). Estimates of the sensitivity, specificity and PPV of UAT using continuous qPCR Ct results were similar to those generated from the model using qualitative qPCR results (Table 2).

**Fig. 2:**
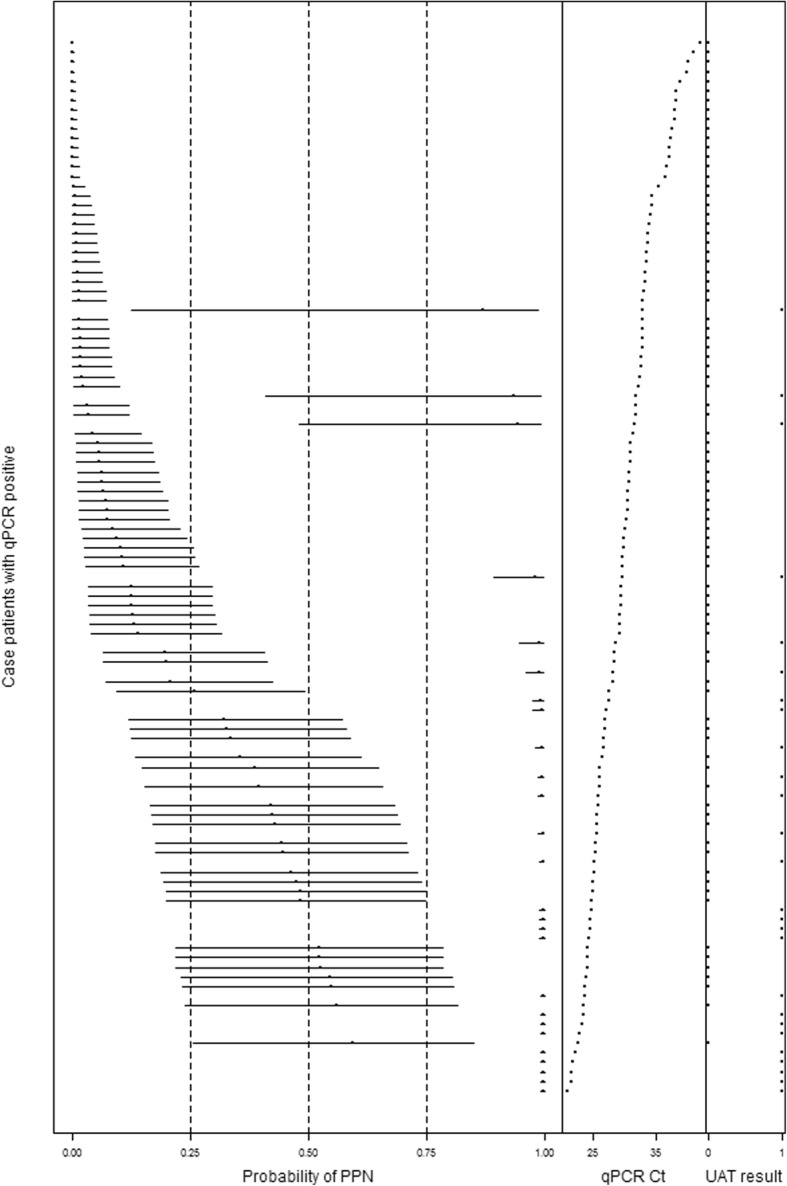
Individual probability of pneumococcal pneumonia (PPN) given pneumococcal Ct value from real-time polymerase chain reaction (qPCR) and urine antigen test (UAT) results in qPCR positive case-patients (*n* = 111)

Based on estimates of individual probability among qPCR-positive cases, the probability for a case-patient having pneumococcal pneumonia ranged from 0 (95% CrI 0–0%) with a Ct value of 42 and a negative UAT result to 100% (95% CrI 99–100%) with a Ct value of 20.9 and a positive UAT result (Fig. 2). The one case-patient who was qPCR negative and UAT positive had a probability of pneumococcal pneumonia of 49% with a wide range of uncertainty (95% CrI: 3–92%).

### Sensitivity analysis

After widening the possible ranges of prior values for parameters derived from controls while keeping the same mean values, the prevalence of pneumococcal pneumonia was estimated as 11% (95% CrI: 7–17%) in the model using binary qPCR results and 11% (95% CrI: 7–16%) for the model using continuous Ct values.

## Discussion

In this analysis, we estimated the prevalence of pneumococcal pneumonia among adults hospitalized with CAP in Thailand and compared the estimates between conventional analyses and BLCMs. Incorporating qPCR Ct values for pneumococcal DNA in NP/OP specimens allowed the probability of pneumococcal pneumonia in individual patients to also be estimated. Our study demonstrated the impact of integrating results from multiple assays and accounting for imperfect test performance on the estimated probability and prevalence of pneumococcal pneumonia.

The prevalence estimated from BLCM was > 25% higher than the prevalence estimated from the primary conventional analysis (10% or 11% vs. 8%). Recent investigations of pneumococcal pneumonia have attempted to take imperfect test performance into account during analysis, due to increased evidence of the imperfectness of the usual reference tests such as UAT and blood culture [25]. The adjusted conventional analysis, although simply based on a fixed value of sensitivity and specificity of UAT, here provided a similar prevalence estimate and uncertainty intervals as the results from BLCM. However although not the case here, conventional analyses may provide potentially misleading estimates of disease prevalence since they neither take into account the uncertainty about these fixed values nor easily incorporate results from multiple imperfect tests. In BLCM, estimates of test performance gathered from the literature as well as test results from controls can be incorporated and quantified as prior distributions. Therefore, the prevalence estimate from BLCM is more robust and utilizes the comprehensive information provided by the case control study design. Compared to prevalence estimates based on conventional analyses using UAT in Thailand (3–4%) [18, 26], our estimates using BLCM or conventional analyses indicate a higher prevalence of pneumococcus in adults in Thailand than previously documented.

Using qPCR Ct values allowed estimation of the probability of pneumococcal pneumonia for each individual case-patient (Fig. 2). In contrast the use of qualitative qPCR values estimated the PPV for pneumococcal pneumonia for all qPCR positive case-patients to be 31% (95% CrI: 20–44%) (Table 2). As has been seen in previous studies, higher density of pneumococcus in the upper airway, as indicated by a lower Ct value, was associated with a higher likelihood of pneumococcal pneumonia [9–13].

The prevalence point estimate and 95% CrIs were similar for the BLCM using binary qPCR and continuous qPCR results when UAT results were included. This finding suggests, at a population level, a much greater predictive value of incorporating UAT testing for pneumococcal pneumonia than the added value of utilizing qPCR Ct values rather than binary qPCR results. For individual case-patients with lower qPCR Ct values and a positive UAT or higher Ct values and negative UAT results, 95% CrIs for the probability of pneumococcal pneumonia were narrow, indicating high certainty in the point estimates. In our study, the addition of the UAT result was necessary to determine pneumococcal pneumonia with a reasonable degree of certainty.

However, the potential for misclassification of disease status by UAT remained as demonstrated by the estimated ~ 17% likelihood that the PPV of UAT is < 90% in BLCM. The specificity of UAT by BLCM was estimated as close to the prior estimate of 100% (99.5%; 98.6–99.9%) but there remained substantial uncertainty around the sensitivity estimates, which ranged from 51 to 83%. These estimates were not updated much compared to the range (50–90%) selected for prior sensitivity of UAT [22] [2, 27–31]. This may indicate that our study data did not add significant new information regarding the limited known sensitivity of UAT. This wide uncertainty range for the sensitivity of UAT supports not utilizing a fixed value for UAT sensitivity to estimate disease prevalence, as would be the case when simply adjusting results of conventional analysis by a set value to account for imperfect sensitivity.

BLCM can be useful to obtain the best possible estimates [23] despite having multiple unknown parameters; however, the posterior estimates from these models depend heavily on the prior distributions of these parameters used. Our carefully designed case-control study provided an important source of information for some prior distributions. To ascertain the robustness of the estimates to this assumption, we performed a sensitivity analysis to account for the possibility that qPCR or UAT results differed between controls and non-pneumococcal pneumonia case-patients; however, the prevalence estimates and all other parameters were almost identical to the main analyses (data not shown). Our estimates of prevalence from BLCM could also be further improved with better knowledge of the sensitivity of urinary antigen assay for disease detection. Recently, the use of a serotype-specific urinary antigen detection assays was demonstrated to substantially increase the detection of pneumococcal pneumonia among adult patients with CAP in the U.S. [25], suggesting the sensitivity of the conventional UAT might be more limited than previously appreciated. Further efforts to more precisely define the true sensitivity of the UAT would be beneficial to provide better prior distributions to be used in future Bayesian analyses. Another limitation was the assumption that the results from qPCR and UAT were conditionally independent of the true pneumococcal pneumonia status. If the test results were conditionally dependent, additional parameters such as correlation coefficients between the sensitivities and specificities of the tests would need to be incorporated into the model to generate reasonable estimates. An inherent limitation in qPCR testing of upper respiratory specimens is the inability to control for the quantity of specimen on each swab. The qPCR-positive swabs with more voluminous specimen could have lower Ct values even if the pathogen density was not actually higher than in a patient whose swab had lower volume. The density of host DNA offers some indication of specimen volume, and in our data, the host DNA Ct values varied little: the mean Ct value among case-patients was 24 (SD: 1.7) and among controls was 24 (SD: 1.4). Therefore, we did not attempt to control for specimen volume with the human DNA Ct. The potential influence of prior antibiotic use on pneumococcal pneumonia estimates is of interest but was not able to be examined in the BLCM models due to the relatively small number of case-patients who had not previously received antibiotics in our study population.

## Conclusions

Using BLCM to integrate results from two independent assays and accounting for imperfect test performance, we found that 11% (95% CrI: 7–17%) of CAP among adults in Thailand was associated with pneumococcus, a higher prevalence than previously estimated from a similar population. Our findings support the hypothesis that upper airway pneumococcal DNA density as based on qPCR Ct value is associated with pneumococcal pneumonia in adults but requires the inclusion of an additional specific pneumococcal test to accurately estimate disease status and prevalence. BLCM can help obtain superior estimates of population disease status when multiple independent test results exist in the absence of a true gold standard reference test in order to inform vaccine cost effectiveness analyses and other disease prevention strategies.

## Additional file

Additional file 1: **Appendix.** Bayesian latent class modeling (BLCM). The Bayesian latent class model, prior selection and sensitivity analyses used in this manuscript. (DOCX 34 kb)

## Abbreviations

BLCM: Bayesian latent class model
CAP: community acquired pneumonia
CDC: Centers for Disease Control and Prevention
CI: confidence interval
CrI: credible interval
Ct: cycle threshold
NP: nasopharyngeal
OP: oropharyngeal
PCR: polymerase chain reaction
PPN: pneumococcal pneumonia
PPV: positive predicative value
qPCR: real time polymerase chain reaction
SARI: severe acute respiratory illness
SD: standard deviation
TNA: Total nucleic acid
UTM: universal transport medium
