# Supplementary material for: Pneumococcal pneumonia prevalence among adults with severe acute respiratory illness in Thailand - comparison of Bayesian latent class modeling and conventional analysis
Source: BMC Infect Dis. 2019 May 15;19:423. doi: 10.1186/s12879-019-4067-3 (PMC6521483; doi:10.1186/s12879-019-4067-3)
Supplement: Supplementary file 1 — Appendix. Bayesian latent class modeling (BLCM). The Bayesian latent class model, prior selection and sensitivity analyses used in this manuscript. (DOCX 34 kb) [file 12879_2019_4067_MOESM1_ESM.docx]

**Appendix: Bayesian latent class modelling (BLCM)**

As in all Bayesian analyses, the BLCM contained two sources of information, prior distributions across all parameters of interest which contain what is known prior to the current study, and the information in the current data set as represented by likelihood function. The parameters were: pneumococcal pneumonia prevalence, distributions of Ct values from both qPCR among pneumococcal pneumonia and non-pneumococcal pneumonia cases, proportions of negative results from qPCR and sensitivity and specificity of the qualitative qPCR and UAT tests. Posterior probabilities and 95% credible intervals (CrI) were calculated for those parameters by combining information in the prior and likelihood function through Bayes Theorem. For model specifications for qualitative qPCR results and UAT, see Joseph *et al.* [[23](#_ENREF_23)]. A free program, BayesDiagnosticTests, is available from http://www.medicine.mcgill.ca/epidemiology/Joseph/software/Diagnostic-Testing.html. Utilizing qPCR Ct values, a model based on Weichenthal *et al.* [[24](#_ENREF_24)] was created. The positive predictive value (PPV) of qPCR and UAT and the probability of pneumococcal pneumonia in individual CAP cases could be derived by posterior estimates of those parameters.

Prior selection

Two general types of prior information were used, informative and non-informative priors. The informative priors were based on information in the data from our control population. The plausible ranges of the non-informative priors were chosen based on current literature review or knowledge and experience from the subject matter experts.

The control population was healthy people without pneumococcal pneumonia as per the description given in the study population section in this manuscript. Therefore, the qPCR and UAT tests results for the controls could be similar to that of non-pneumococcal pneumonia in case- patients. With this assumption, we derived the following informative priors. There were 57 qPCR positive results among the 217 controls and these Ct values were distributed with a mean of 33 and SD of 4.8. The specificity of qualitative qPCR and proportion of negatives in non-pneumococcal pneumonia in the continuous qPCR model were derived by using *Beta*(1, 1) conjugated with *Binomial*(160, 57), hence the posterior a *Beta*(161, 58). The mean Ct value with Normal (33.5, 0.65) and standard deviation with *Uniform* (4, 6) of Ct values in non-pneumococcal pneumonia in case-patients were estimated from the distribution of Ct values in 57 qPCR positive patients. There was 1 UAT positive among 217 controls, the specificity of UAT was derived by using *Beta*(1, 1) conjugated *Binomial* (216, 1), hence the posterior a *beta*(217, 2) (Table 1).

As pneumococcal pneumonia prevalence was the primary parameter of interest, a non-informative range from 0 to 100% was selected then converted to the beta distribution, *Beta* (1, 1). There is limited information about the sensitivity of qPCR for diagnosing pneumococcal pneumonia [[34](#_ENREF_34)], we therefore selected a non-informative range from 0 to 100% and converted this range to the beta distribution, *Beta* (1,1). Plausible values for the sensitivity of UAT were selected to be 50-90% based on previous studies [2, 22, 27-32] and converted to the *Beta* distribution (14, 6). Given limited prior information, the range 0-100% was selected and converted to the *Beta* (1,1) for the proportion of negative results from qPCR similar to what was done for qPCR sensitivity. The *Normal* (30, 5) was used for the mean Ct value and the *Uniform* (0, 5) for the SD of the Ct values. We believed that *Normal* (30, 5) with variability of *Uniform* (0, 5) covered all plausible Ct values in pneumococcal pneumonia (Table 1).

Table 1. Priors for prevalence, sensitivities and specificities of qualitative qPCR and UAT, means and standard deviations of qPCR Ct values.

|  | **Binary qPCR model** | | | **Continuous qPCR model** | | |
| --- | --- | --- | --- | --- | --- | --- |
|  | Range | Distribution | Wider priors from controls for sensitivity analysis | Range | Distribution | Wider priors from controls for sensitivity analysis |
| **Prevalence** | 0 – 100% | *Beta* (1,1)* |  | 0 – 100% | *Beta* (1, 1)* |  |
| **Sensitivity of qPCR** | 0 – 100% | *Beta* (1,1)* |  |  |  |  |
| **Specificity of qPCR** |  | *Beta* (161, 58)^#^ | *Beta*(40.25,14.5) |  |  |  |
| **Proportion of negative qPCR in PPN** |  |  |  | 0 – 100% | *Beta* (1, 1)* |  |
| **Proportion of negative qPCR in Non-PPN** |  |  |  |  | *Beta* (161, 58)# | *Beta*(40.25,14.5) |
| **Mean Ct of qPCR among PPN** |  |  |  |  | *Normal* (30, 5)* |  |
| **Mean Ct of qPCR among Non-PPN** |  |  |  |  | *Normal* (33.05, 0.65)# | *Normal*(33.05, 1.3) |
| **SD of Ct of qPCR among PPN** |  |  |  |  | *Uniform* (0, 5)* |  |
| **SD of Ct of qPCR among Non-PPN** |  |  |  |  | *Uniform* (4, 6)# |  |
| **Sensitivity of UAT** | 50 – 90% | *Beta* (14, 6)* |  | 50 – 90% | Beta (14, 6)* |  |
| **Specificity of UAT** |  | *Beta* (217,2)^#^ | *Beta*(43.4,2) |  | *Beta* (217,2)# | *Beta*(43.4,2) |

**Beta* (α, β): a probability distribution for a parameter that takes the values in the interval [0-1]. The coefficients α and β, for each coefficient, was derived by matching the center of the range with the mean of the beta distribution given by α/(α+β), and matching the standard deviation of the beta distribution, given by
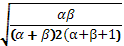

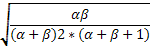
$\sqrt{\frac{\alpha*\beta}{{(\alpha+\beta)}^{2}*(\alpha+\beta+1)}}$, with one quarter of the total range [[23](#_ENREF_23)]. *Normal* (mean, SD): a bell curve probability distribution for a parameter that takes the values centered on the mean with SD. *Uniform* (a, b): an equal probability distribution for a parameter that takes the values from a to b.

#Distribution was derived from controls.

WinBUGS program

# Probability of 'success' for variable 'dichot' are

# dichot.p[1] in Unexposed group (1-specificity)

# dichot.p[2] in Exposed group (sensitivity)

# each with prior distrns Beta(dichot.p.alpha[i], dichot.p.beta[i]), i=1,2, to be provided in data file

model

{

prev ~ dbeta(prev.alpha, prev.beta)

for (i in 1:Unclassified)

{

exposed[i] ~ dbern(prev)

}

for (i in 1:N)

{

group[i] <- 1 + exposed[i]

}

for (g in 1:2)

{

stp.num.PNull[g] ~ dbeta(stp.num.PNull.alpha[g], stp.num.PNull.beta[g])

dichot.p[g] ~ dbeta(dichot.p.alpha[g], dichot.p.beta[g])

uat[g]<- 1-dichot.p[g]

}

for (i in 1:Unclassified)

{

binom.stp.num[i] ~ dbin(stp.num.PNull[group[i]], 1)

dichot.m[i] ~ dbin(dichot.p[group[i]], 1)

}

for (i in 1:N.stp.num)

{

x.stp.num[i] ~ dnorm(stp.num.mu[group[idno.stp.num[i]]], stp.num.prec[group[idno.stp.num[i]]])

}

for (g in 1:2)

{

stp.num.sd[g] ~ dunif(stp.num.sd.lower[g], stp.num.sd.upper[g])

stp.num.prec[g] <- pow(stp.num.sd[g], -2)

stp.num.mu[g] ~ dnorm(stp.num.mu.mean[g], stp.num.mu.prec[g])

stp.num.mu.prec[g] <- pow(stp.num.mu.sd[g], -2)

}

one.stp.num <- 1

one.stp.num ~ dbern(one.stp.num.cond)

one.stp.num.cond <- step(stp.num.mu[1] - stp.num.mu[2])

for (i in 1:Unclassified)

{

for (g in 1:2)

{

logLikelihood[i, g] <- stp.num.count[i] / 2 * log(stp.num.prec[g])

- stp.num.count[i] * pow(x.complete.stp.num[i] - stp.num.mu[g], 2) * stp.num.prec[g] / 2

+ m.stp.num[i] * log(stp.num.PNull[g])

+ (n.stp.num[i]-m.stp.num[i]) * log(1-stp.num.PNull[g])

+ dichot.m[i]*log(dichot.p[g]) + (1-dichot.m[i])*log(1-dichot.p[g])

}

ExposureProb[i] <- 1 / (1 + exp(logLikelihood[i, 1] + log(1-prev) - logLikelihood[i, 2] - log(prev)))

}

PPV.uat <- prev*dichot.p[2]/(prev*dichot.p[2]+(1-prev)*dichot.p[1])

Prob_90pctlower<-step(0.9-PPV.uat)

# ---- Estimation of ROC curve at roc.point's -------

for (i in 1:26)

{

roc.sens[i] <- phi((roc.point[i]-stp.num.mu[2])/stp.num.sd[2])

roc.spec[i] <- 1 - phi((roc.point[i]-stp.num.mu[1])/stp.num.sd[1])

one.minus.roc.spec[i] <- phi((roc.point[i]-stp.num.mu[1])/stp.num.sd[1])

}

}

Sensitivity analyses

We performed a sensitivity analysis by keeping the same means but widening the ranges for the specificity of qualitative qPCR, mean Ct values and proportion of negatives in the continuous qPCR model among among non-PPN cases. For the specificity of UAT, the range could only be widened towards the lower bound as the upper bound already reached 100% (Table1). The same two-test models for either qualitative or qPCR Ct values with UAT were then run to check how the parameters, especially the prevalence of pneumococcal pneumonia, changed.
